# On the structure and oxygen transmission rate of biodegradable cellulose nanobarriers

**DOI:** 10.1186/1556-276X-7-192

**Published:** 2012-03-19

**Authors:** Gary Chinga-Carrasco, Kristin Syverud

**Affiliations:** 1 Paper and Fibre Research Institute (PFI), Høgskoleringen 6b, Trondheim, 7491, Norway

**Keywords:** Nanoparticles, Polymers, Porous materials, Cellulose, Films

## Abstract

Cellulose nanofibrils have been proposed for novel barrier concepts, based on their capability to form smooth, strong and transparent films, with high oxygen barrier properties. A series of cellulose-based films were manufactured and tested with respect to their oxygen transmission rate (OTR) capabilities. The obtained OTR levels were considerably better than the levels recommended for packaging applications. Part of the nanofibrillated material applied in this study was produced with 2,2,6,6-tetramethylpiperidinyl-1-oxyl (TEMPO) mediated oxidation as pretreatment. Films made of TEMPO-pretreated samples yielded lower OTR values. The minimum obtained OTR value was 3.0 mL m^-2 ^day^-1 ^atm^-1 ^with a corresponding oxygen permeability of 0.04 mL mm m^-2 ^day^-1 ^atm^-1^, tested at 50% relative humidity. The good barrier properties are due to the compact and dense structure of the films, as revealed by field-emission scanning electron microscopy. A relationship between OTR and the structure of the corresponding nanofibril-based films was confirmed.

## Background

Cellulose nanofibrils are nano-components of a cellulosic material produced through a fibrillation process [1]. Several applications have been envisaged for cellulose nanofibrils due to their particular rheological, optical and strength properties [2-6]. The materials have also been proposed for packaging applications, based on their capability to form smooth, strong and transparent films, with high oxygen barrier properties [5,7-10]. However, films made of cellulose nanofibrils are hydrophilic, having low moisture barrier properties. Surface modification has thus been proposed for reducing water wettability [7,10,11], which may be necessary in food packaging applications.

The density of nanofibril-based films is an important property to quantify, considering their potential application as barriers in packaging. The density gives an indication of the consolidation of the films during production. The more consolidated a given film is, the larger the density. Yang et al. [12] reported a relationship between the oxygen transmission rate (OTR) and the density of regenerated cellulose films. The intrinsic thickness of relatively thin films is an essential measure for estimating the density of the material. The thickness of cellulose films can be measured with electron microscopy techniques, as reported recently [13]. In addition, the crystallinity degree of a given cellulose material has been reported to affect the OTR [5,8], i.e. increasing the crystallinity degree reduces the oxygen permeability.

The purpose of this study is to shed light on the structure of nanofibril-based films and on how this complex structure limits the oxygen transmission rate through the material. A relationship between the structure of a series of nanofibril-based films and the corresponding OTR levels is confirmed.

## Methods

A series of nanofibril qualities were utilized in this study. The nanofibril qualities were produced from *Eucalyptus *and *Pinus radiata*, as described by Syverud et al. [14]. Some of the pulp fibres were chemically pretreated, according to Saito et al. [15]. 2,2,6,6-Tetramethylpiperidinyl-1-oxyl (TEMPO) was applied to catalyse the oxidation of primary alcohol groups using NaClO. TEMPO-mediated oxidation facilitates a homogeneous fibrillation. A more detailed description is given by Syverud et al. [14].

The kraft pulp fibres (0.5% consistency) were homogenized with a Rannie 15 type 12.56X homogenizer (APV, SPX Flow Technology, Silkeborg, Denmark), operated at 1,000 bar pressure. The fibrillated materials were collected after three and five passes through the homogenizer (Table 1).

**Table 1:** Films were made from each series of the fibrillated materials

Series	Fibre	Pretreatment	Homogenization (# passes)
F01	*Eucalyptus*	-	3

F02	*P. radiata*	-	3

F03	*Eucalyptus*	TEMPO	3

F04	*P. radiata*	TEMPO	3

F05	*Eucalyptus*	-	5

F06	*P. radiata*	-	5

F07	*Eucalyptus*	TEMPO	5

F08	*P. radiata*	TEMPO	5

'# passes' indicates the number of passes the cellulose material passed through the homogenizer. Generally, the more passes through the homogenizer the more fibrillated the material is

Films (F01 to F08) were prepared in plastic petri dishes by free drying. The drying temperature was 23°C. Films of samples F01, F02 and F07 were additionally made in a cylindrical mould [5]. The films made in the cylindrical mould are considered to be prepared in restrained conditions due to the supporting wire system. Film F07, made under restrained conditions, was additionally dried at 105°C for 2 h.

A scanning electron microscopy (SEM) cross-sectional analysis (thickness and roughness) was performed in backscatter electron imaging (BEI) mode as described by Chinga-Carrasco et al. [13]. The applied microscope was a Hitachi S-3000 variable pressure SEM (Hitachi High-Technologies Corporation, Minato-ku, Tokyo, Japan), using a solid state backscatter detector. The densities of the films (*δ*_f_) are given by the relationship *γ/τ*_f_, where *γ *and *τ*_f _correspond to the grammage and mean thickness of the films, respectively.

A surface structural quantification based on laser profilometry (LP; Lehmann, Lehman Mess-Systeme AG, Baden-Dättwil, Germany) and atomic force microscopy (AFM; Nanoscope Dimension 3100 controller, Digital Instruments-Vecco, Santa Barbara, CA, USA) was performed on the films, as described by Chinga-Carrasco et al. [13] and Syverud et al. [14]. Ten local areas per sample were assessed with LP. The size of the local areas was 2 × 2 mm^2^, with a lateral resolution of 1 μm. The AFM analysis was performed on local areas of 5 × 5 μm^2^, with a lateral resolution of 10 nm.

The embedded films were exposed to a small quantity of water to induce a delamination of the cross-sectional structure. After drying, the samples were then covered with a conductive layer. Images were acquired at various magnifications with a field-emission SEM (Zeiss Ultra field-emission SEM, Carl Zeiss AG, Oberkochen, Germany). The images were acquired in secondary electron imaging (SEM-SEI) mode. In addition, a field-emission SEM analysis, applying the inlens detector, was performed for revealing the surface nanostructure of the films.

The OTR was measured with a Mocon OX-TRAN^® ^1/50 test system (Mocon, Minneapolis, MN, USA) at 50% relative humidity and 23°C.

## Results and discussion

**Figure 1:**
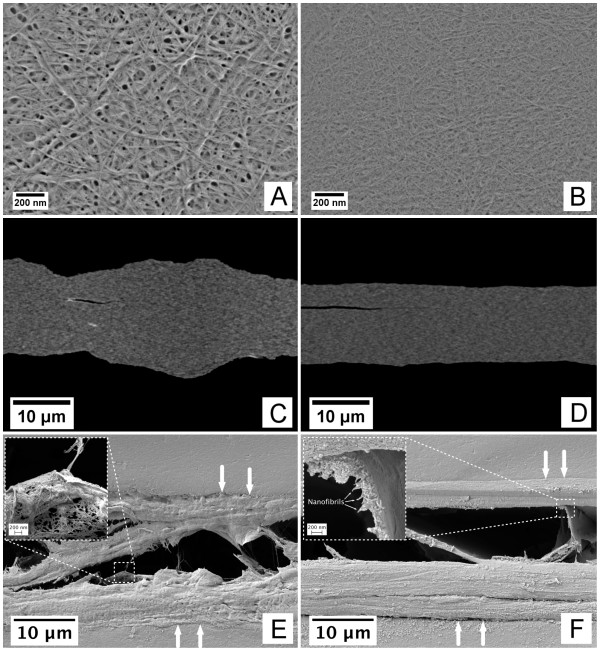
**Structure of nanofibril-based films**. (**A**) and (**B**) correspond to the surface structures of films F02 and F08, visualized in field emission SEM. (**C**) and (**D**) correspond to the cross-sectional structures (F01 and F04) visualized in SEM-BEI mode. The images have been enhanced for visualization purposes. The small dark cracks observed in images (C) and (D) are most probably due to the preparation for SEM analysis. (**E**) and (**F**) correspond to the cross-sectional structures after being subjected to water. The white arrows indicate the *z*-direction of the films and the boundaries of the upper and lower sides. The insets in (E) and (F) indicate some lamellar structures exemplifying the delamination of the films.

Considering the fibrillation degree of the material, where the major fraction corresponds to cellulose nanofibrils, suggests that the films have low porosities and high densities (Figure 1A, B, C, D). The films are thus very compact (Figure 1C, D). The films were exposed to a small quantity of water to provoke a partial delamination in the *z*-direction of the films (Figure 1E, F). The opening of the films in the *z*-direction is particularly interesting. The films appear to be composed of a series of layers, forming defined lamellae [16]. The films composed of fibrils with a relatively broad size distribution delaminate more chaotically than the films composed of homogeneous fibril sizes. Despite the evidenced differences of the delamination patterns, both films presented in Figure 1 show defined nanofibril layers (Figure 1E, F (insets)). Note that the layers are formed by randomly positioned fibrils, creating pore structures even in the middle layers of the films (Figure 1E, inset). Considering that the films are composed of layered structures indicates that the pore structure is not continuous, i.e. the pore structure seems to have high tortuosity and low pore connectivity.

OTR values lower than 20 mL m^-2 ^day^-1 ^have been recommended for packaging applications [17]. The OTR values of the films assessed in this study were between 3.0 and 4.4 mL m^-2 ^day^-1 ^atm^-1^. The lowest OTR level was 3.0 mL m^-2 ^day^-1 ^atm^-1^, which corresponds to an oxygen permeability of 0.04 mL mm m^-2 ^day^-1 ^atm^-1^. This is a good indication of the good barrier properties of the films, which are comparable to EVOH (3 to 5 mL m^-2 ^day^-1 ^atm^-1^) [17] and cellophane (3 mL m^-2 ^day^-1 ^atm^-1^) [18]. For a comprehensive overview of the OTR levels of some renewable and synthetic polymers, see the study of Aulin et al. [8].

It is worth to notice that despite the major differences between the assessed films with respect to the nanofibril morphology and pore structure (Figure 1), the OTR values differ only by roughly 0.5 to 1.0 mL m^-2 ^day^-1 ^atm^-1^. This seems to confirm the poor connectivity of the pores in the film structures.

**Figure 2:**
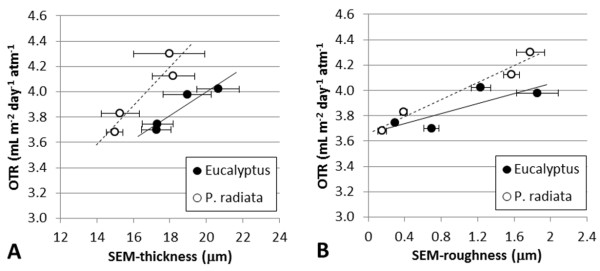
**SEM structural analysis**. (**A**) SEM thickness. (**B**) SEM roughness. The average values are given with the corresponding 95% confidence interval.

**Figure 3:**
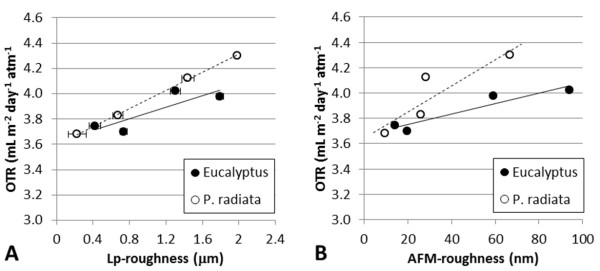
**Surface quantification**. (**A**) Surface roughness as measured with LP. (**B**) Surface roughness as measured with AFM. The average values are given with the corresponding 95% confidence interval.

For a given grammage, the more porous a structure is, the thicker the corresponding film. OTR values can thus be related to the corresponding film thicknesses, as exemplified in Figure 2. Films with relatively high porosity are composed of relatively thick fibrils and poorly fibrillated fibres. The poorly fibrillated fibres are expected to influence the corresponding micro-roughness of the films. This is confirmed in this study by two independent methods, i.e. SEM and laser profilometry (Figures 2B and 3A). Note that even AFM, which assesses the structure at the nano-level, reveals the same trends, i.e. increasing the roughness increases the OTR (Figure 3B). The positive relationship between roughness and OTR is considered a confirmation of the adequacy of microstructural analysis for understanding the structure of nano-engineered cellulose films.

**Figure 4:**
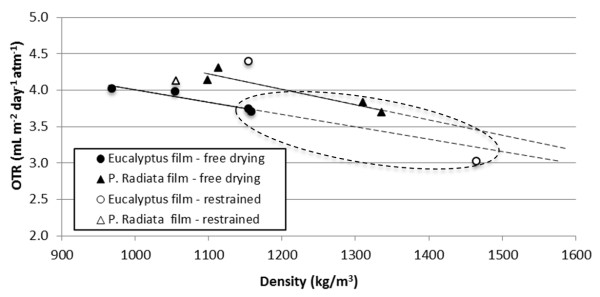
**OTR as a function of density for films with a grammage of 20 g/m^2^**. OTR was measured at 50% relative humidity. The measurements corresponding to the TEMPO-pretreated samples have been encircled.

Considering the positive correlations between a given film structure and the corresponding OTR levels suggests that increasing the density decreases the OTR. This is confirmed in Figure 4. It is worth to note that films made of TEMPO-pretreated samples (F03, F04, F07, F08) yield higher density and lower OTR values. This is due to the highly fibrillated material, composed mostly of nanofibrils (< 20 nm) and which forms compact and low-porosity structures.

The *Eucalyptus *nanofibril-based films, made by free drying, yield lower densities than the corresponding *P. radiata *nanofibril-based films. One thus expected higher OTR values for the *Eucalyptus *nanofibril-based films. *Eucalyptus *films yield, however, similar OTR values at lower density (apparent higher porosity) compared to *P. radiata *(Figure 4, solid symbols). It was speculated whether the apparent lower density of the films, based on *Eucalyptus *nanofibrils, was caused by a relatively high shrinkage degree, as quantified by Syverud et al. [14]. Shrinkage leads to higher mass per unit size. This thus leads to an underestimation of the quantified density, as the local grammage will be higher than the target grammage (20 g/m^2^). The density-OTR correlations should probably be translated to higher density levels, approaching the OTR-density correlation of the *P. radiata *nanofibril-based films. Films made under restrained conditions were analysed to verify this assumption. The OTR-density values of the films made under restrained conditions yielded variable results, probably due to the uneven structure caused by the formation procedure. However, the OTR-density measurements are in the same range as the measurements of the free-dried films and follow the same trend, i.e. increasing density reduces the OTR. This is a confirmation of the expected OTR-density relationship.

## Conclusions

The obtained OTR levels of the films used in this study (grammage 20 g/m^2^) were considerably better than the levels recommended for packaging applications. Films made of TEMPO-pretreated samples yielded lower OTR values. The minimum obtained OTR was 3.0 mL m^-2 ^day^-1 ^atm^-1 ^with a corresponding oxygen permeability of 0.04 mL mm m^-2 ^day^-1 ^atm^-1^, tested at 50% relative humidity. The good barrier properties are due to the compact and dense structure of the films. A relationship between OTR and the structure of the corresponding nanofibril-based films was confirmed.

## Abbreviations

AFM: atomic force microscopy; BEI: backscatter electron imaging; LP: laser profilometry; OTR: oxygen transmission rate; SEI: secondary electron imaging; SEM: scanning electron microscopy; TEM: transmission electron microscopy; TEMPO: 2,2,6,6-tetramethylpiperidinyl-1-oxyl.

## Competing interests

The authors declare that they have no competing interests.

## Authors' contributions

GC-C was involved in the production and characterisation of cellulose nanofibrils; performed the LP, SEM and FESEM analyses; wrote the manuscript and performed the corresponding revisions. KS was involved in the production and characterisation of cellulose nanofibrils, was responsible for the oxygen transmission rate measurement and, has been involved in revising the manuscript critically for important intellectual content. All authors read and approved the final manuscript.
